# Small heat shock protein 20 (Hsp20) facilitates nuclear import of protein kinase D 1 (PKD1) during cardiac hypertrophy

**DOI:** 10.1186/s12964-015-0094-x

**Published:** 2015-03-07

**Authors:** Yuan Yan Sin, Tamara P Martin, Lauren Wills, Susan Currie, George S Baillie

**Affiliations:** Institute of Cardiovascular and Medical sciences, CMVLS, University of Glasgow, Glasgow, G128QQ UK; Strathclyde Institute of Pharmacy & Biomedical Sciences, University of Strathclyde, Hamnett building, 161 Cathedral Street, Glasgow, G4 ORE UK

**Keywords:** Hsp20, PKD1, Cardiac hypertrophy, Peptide array

## Abstract

**Background:**

Nuclear import of protein kinase D1 (PKD1) is an important event in the transcriptional regulation of cardiac gene reprogramming leading to the hypertrophic growth response, however, little is known about the molecular events that govern this event. We have identified a novel complex between PKD1 and a heat shock protein (Hsp), Hsp20, which has been implicated as cardioprotective. This study aims to characterize the role of the complex in PKD1-mediated myocardial regulatory mechanisms that depend on PKD1 nuclear translocation.

**Results:**

In mapping the Hsp20 binding sites on PKD1 within its catalytic unit using peptide array analysis, we were able to develop a cell-permeable peptide that disrupts the Hsp20-PKD1 complex. We use this peptide to show that formation of the Hsp20-PKD1 complex is essential for PKD1 nuclear translocation, signaling mechanisms leading to hypertrophy, activation of the fetal gene programme and pathological cardiac remodeling leading to cardiac fibrosis.

**Conclusions:**

These results identify a new signaling complex that is pivotal to pathological remodelling of the heart that could be targeted therapeutically.

## Introduction

Cardiac myocytes respond to pathological stress via activation of the fetal gene programme (reviewed in [1]). Essential to this process, is the activation of cardiac protein kinase D (PKD). PKD1 is a ubiquitously expressed serine/threonine kinase and is the most characterised member of a kinase family within the CaMK group (comprising PKD1, PKD2, and PKD3) of kinases [2]. In the adult heart, PKD1 is activated in response to chronic adrenergic signalling, hypertension and pressure overload. Phosphorylation and hence activation of PKD1 occurs initially via binding to diacylglycerol [3] at its cystein-rich domain with kinase activation/translocation being triggered by PKC-phosphorylation and the release of pleckstrin homology (PH)-mediated PKD1 inhibition [4]. Upon cell stimulation, PKD isoforms translocate from the cytosol to DAG-containing microenvironments at the plasma membrane and then back to the cytosol and into the nucleus [5]. PKD has been implicated in a variety of cellular responses including cell proliferation, inflammation, cardiac contractility and hypertrophy [2]. In the heart, PKD1 has been shown to regulate myofilament function and Ca^2+^ sensitivity by phosphorylating cardiac troponin I [6,7]. PKD1 also regulates cardiac myocyte hypertrophy by translocating into the nucleus and directly phosphorylating class II histone deacetylase 5 (HDAC5) [8]. HDACs deacetylate histones resulting in transcriptional repression. HDAC phosphorylation, for example by PKD1 and CaMKII [9], creates docking sites for 14-3-3 chaperone proteins that escorts them from the nucleus to the cytoplasm, thus relieving downstream transcription factors (such as myocyte enhancer factor-2 (MEF2)), of their repression [10,11]. However, little is known about the molecular events that govern PKD1 nuclear translocation.

In a proteomics screen to discover novel partners for the chaperone heat shock protein 20 (Hsp20), we found PKD1 to be a robust Hsp20-binding protein. Hsp20 (reviewed in [12]) displays cardioprotective actions including anti-ischaemic, anti-apoptotic and anti-hypertrophic effects. At basal level, HSP20 is primarily found in the cytosol but a subpopulation may translocate into the nuclear compartment in response to stress signals [13].

In this manuscript, we verify the interaction between Hsp20 and PKD1 and use novel peptide disruptors to show firstly, that formation of the Hsp20-PKD1 complex is required for hypertrophic signaling, secondly, that Hsp20 facilitates the nucleo-cytoplasmic shuttling of PKD1 and thirdly, that the Hsp20-PKD1 interaction promotes stress related left ventricular (LV) dysfunction and cardiac fibrosis.

## Results and discussion

### Hsp20 and PKD1 form a complex in cardiac myocytes

**Figure 1:**
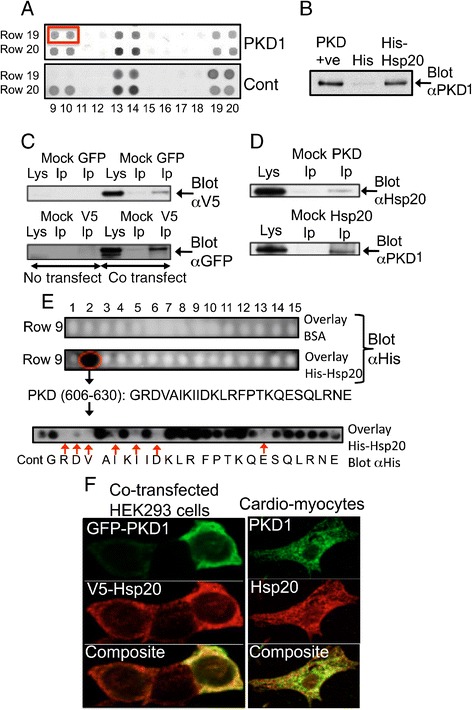
**PKD1 and Hsp20 form a signaling complex in cardiac myocytes. A**. ProtoArray Human Protein Microarray screen identified PKD1 as an interacting partner for Hsp20 (top panel, compared to control, bottom panel). **B**. *In vitro* pull-down assay showed that recombinant His-Hsp20, but not control His-tag, co-purifies with PKD1. **C**. Overexpressed Hsp20-V5 and GFP-PKD1 co-immunoprecipitate when expressed in HEK293 cells. **D**. Endogenous Hsp20 and PKD1 from cardiac myocyte lysate co-immunoprecipitate. Mock IP represents respective IgG only. Data representative of n = 3 independent experiments. **E**. Peptide array analysis identifies Hsp20 binding site within the catalytic domain of PKD1 (G^606^-E^630^). **F**. Overexpressed Hsp20-V5 and GFP-PKD1 co-localise in HEK293 cells and endogenous Hsp20 and PKD1 co-localise in cardiac myocytes. Pearson’s coefficient for Hsp20 and PKD1 is shown along with Manders’ coefficients (M1 and M2), which represent the fraction of the Hsp20 red overlapping with PKD1 green and the fraction of PKD1 green overlapping with the Hsp20 red, respectively. The results are shown as means ± SEM of several images where at least 10 individual cells were analysed for each coefficient.

The protective functions of Hsp20 in a cardiac setting have received much attention and in an attempt to elucidate the molecular mechanisms involved, we endeavored to characterise putative interactors of Hsp20 using high-density ProtoArray Human Protein Microarray (Invitrogen, UK) analysis. Using this method, a previously unknown Hsp20-binder was identified as PKD1 (Figure 1A). The interaction was verified by pull-down analysis of recombinant proteins (Figure 1B) and immunoprecipitation of exogenously (Figure 1C) and endogenously (Figure 1D) expressed cardiac myocyte proteins. Co-localisation immunostaining (Figure 1F) demonstrated positive correlation in HEK293 cells overexpressing Hsp20 and PKD1 (Pearson’s 0.62 ± 0.03, Manders’ 0.81 ± 0.02, M1 0.96 ± 0.01, M2 0.95 ± 0.01) and in myocytes expressing endogenous Hsp20 and PKD1 (Pearson’s 0.81 ± 0.02, Manders’ 0.88 ± 0.01, M1 0.86 ± 0.03, M2 0.93 ± 0.01). This data suggests that Hsp20 and PKD1 locate to the same region of cells. Peptide array technology was also utilized, where the sequence of PKD1 was spotted in 25mer peptides, each shifted by five amino acids [14]. As shown in Figure 1E, Hsp20 interacted directly with PKD1, and the interaction site was mapped to a 25mer sequence spanning residues 606 to 630 within the PKD1 catalytic unit. Subsequent alanine scanning peptide array, whereby every amino acid was replaced sequentially with alanine, pinpointed the residues on PKD1 that are essential for interaction as R607, D608, V609, I611, I613, D615, E624 (Figure 1E lowest panel).

## The Hsp20-PKD1 complex is required for hypertrophic signaling

As both PKD1 and Hsp20 are strongly implicated in hypertrophic signaling [11,13,15], we decided to probe the functionality of the complex by using cell-permeable peptides, designed using the information gleaned from peptide array analysis (Figure 1E), to disrupt the Hsp20-PKD1 interaction. Previous work from our group has shown that cell-permeable analogues of 25mer peptides identified in this manner, often can be used to disrupt signalling complexes, thereby effecting specific cardiovascular-specific functional outputs such as phosphorylation of the β_2_-adrenergic receptor by PKA [16], recruitment of EPAC to the β _2_-adrenergic receptor [17], permeability of the vascular epithelium [18] and tubule formation by human arterial endothelial cells [19]. Indeed, we have already used this approach successfully to disrupt another Hsp20 binding partner, PDE4D5, in order to induce cardioprotection [15].

**Figure 2:**
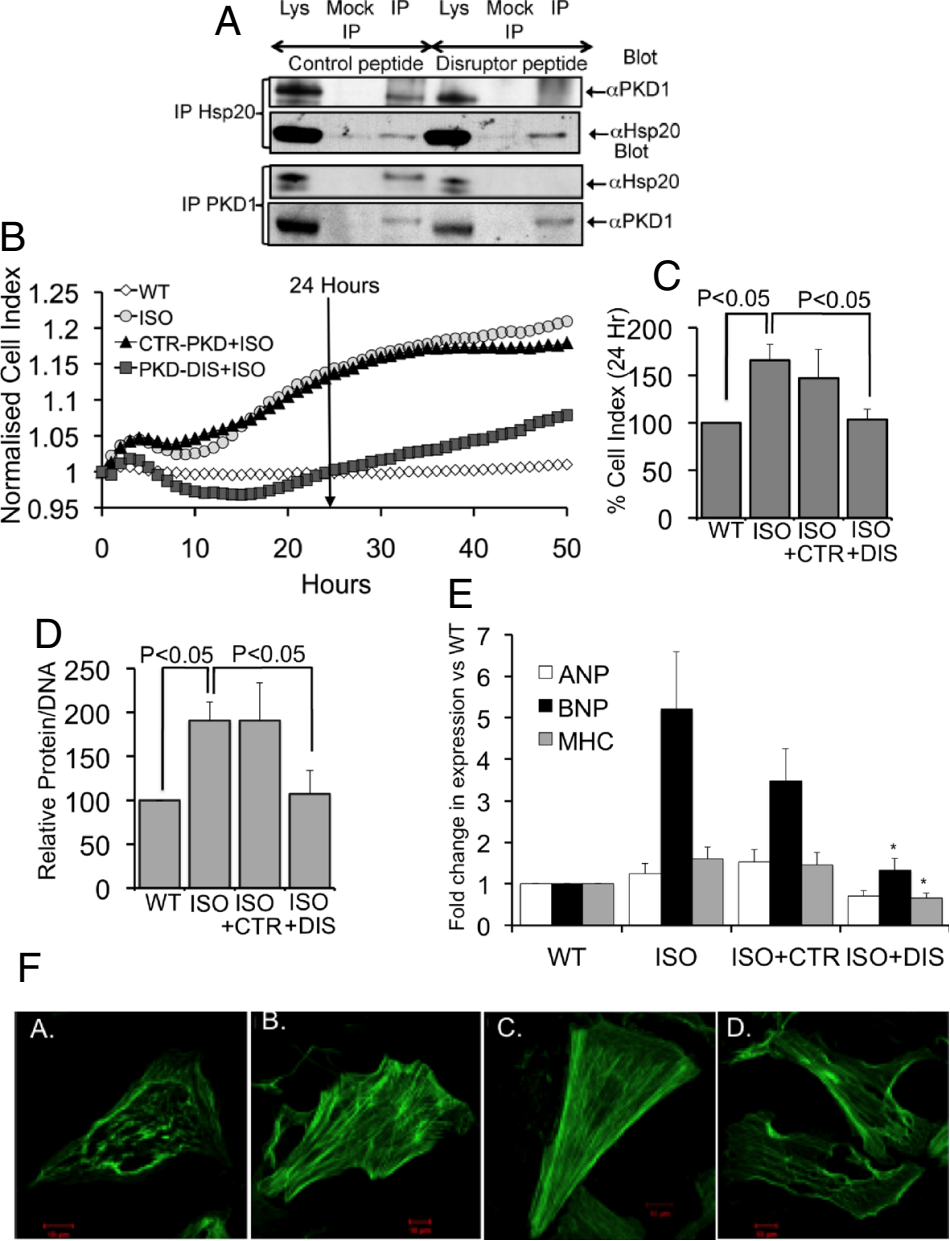
**Disruption of the Hsp20-PKD1 complex attenuates hypertrophic signaling in neonatal cardiac myocytes. A**. A cell permeable disruptor peptide (DIS), but not scrambled control (CTR), ablates interaction of PKD1 and Hsp20 in cardiac myocytes. **B**. and **C**. The Hsp20-PKD1 disruptor peptide reduces cardiac myocyte cell size following chronic β-adrenergic (isoprenaline; ISO) stimulation. **D**. The Hsp20-PKD1 disruptor peptide reduces relative protein/DNA ratio of cells following chronic β-adrenergic stimulation (n = 9). **E**. The Hsp20-PKD1 disruptor peptide reduces the expression of hypertrophic markers atrial natruiretic peptide (ANP), brain natruiretic peptide (BNP) and β-myosin heavy chain (β-MHC) following chronic β-adrenergic stimulation (n = 7). **F**. The Hsp20-PKD1 disruptor peptide reduces actin cytoskeletal rearrangement following chronic β-adrenergic stimulation. Data representative of n = 3 independent experiments.

Gratifyingly, a disruptor peptide comprising of PKD1 residues 606 to 630 (DIS), but not a control peptide (CTR) with three of the seven key residues replaced by alanine (GAAVAIKIIAKLRFPTKQESQLRNE), ablated co-immunopreciptation of endogenous Hsp20 and PKD1 from neonatal cardiac myocyte lysates (Figure 2A). As we could successfully disrupt the Hsp20-PKD1 complex with disruptor peptide (Figure 2A), we were interested to see if it would be able to attenuate the hypertrophic response induced by chronic β-agonist (isoprenaline; ISO) stimulation of neonatal cardiac myocytes. To this end, we utilised 3 different assay methods to measure the effectiveness of the peptide to confer protection against β-adrenergic receptor-triggered hypertrophic signalling. Firstly, we employed a novel approach utilizing xCELLigence technology, which we have previously developed [15] to measure the size of cardiac myocytes (Figure 2B and C). Secondly, we measured the relative protein/DNA ratio of cells (Figure 2D) and thirdly, in order to investigate whether the peptide affected increased fetal gene expression that characterises the hypertrophic response, we determined the mRNA levels of 3 hypertrophy marker genes atrial naturietic peptide (ANP), brain naturietic peptide (BNP) and β-myosin heavy chain (β-MHC) (Figure 2E). All three techniques suggested that the disruptor peptide (but not control peptide) could attenuate the hypertrophic response. Note that differences seen in BNP between ISO and ISO plus control peptide are not significant (p = 0.46) Cell size, measured in real-time by cell index (CI; Figure 2B and C) and protein/DNA ratio (Figure 2D) of cells increased following chronic ISO treatment and were significantly reduced by pre-treatment with disruptor peptide but not with control peptide. The Hsp20-PKD1 disruptor peptide also significantly reduced the upregulation of fetal gene expression (Figure 2E) triggered by chronic β-agonist stimulation. Another key characteristic of hypertrophy is the reorganization of actin filaments [20] and we were able to observe the expected formation of β-adrenergic–stimulated stress fibers when compared with untreated cells (Figure 2F). Interestingly, the actin cytoskeletal rearrangement stimulated by hypertrophy induction was visibly reduced in cells pre-treated with disruptor peptide, but not control peptide, (Figure 2F) suggesting that the Hsp20-PKD1 association is also likely to have the capacity to regulate the integrity of cellular actin filaments.

## Hsp20 acts as a nuclear import chaperone for PKD1

**Figure 3:**
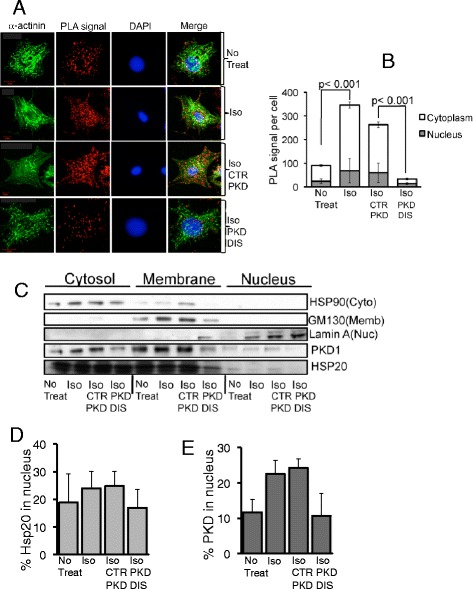
**Hsp20 may act as a nuclear chaperone for PKD1. A** and **B**. A novel proximity ligation assay was used to visualize and quantify PKD1 nuclear entry in neonatal cardiac myocytes. Images are shown as maximum projections of z-stacks of confocal images. PLA signals indicating Hsp20-PKD1 complex formation were quantified using the analyse particles plugin of ImageJ software. For all experiments, quantifications were performed from at least 12 images and expressed as mean number of signals per cell. **C**, **D** and **E**. Cell fractionation techniques were used to visualize and quantify the cellular distribution profile of PKD1 and Hsp20 from cardiac myocytes. The disruptor peptide (PKD DIS) prevented ISO-induced nuclear translocation of Hsp20-PKD1 complexes, compared to control peptide (CTR PKD). Data representative of n = 3 independent experiments.

In normal functioning cardiac myocytes, the majority of PKD1 localises to the cytosol, while a subpopulation is found in various organelles, such as the nucleus, plasma membrane, mitochondria and trans Golgi network [21]. PKD1 is bound by DAG at its cystein-rich domain and kinase activation/translocation is triggered by PKC-phosphorylation and the release of PH-mediated PKD1 inhibition [5]. This chain of molecular events promotes PKD1 activation, and it has been established that PKD1 undergoes nuclear shuttling to initiate gene transcription following chronic β-adrenergic -agonist stimulation [8]. However, little is known about the molecular events that underpin PKD1 nuclear translocation. A-kinase anchoring protein (AKAP-Lbc), a protein that is known to bind PKD1 [22] and Hsp20 [23], can coordinate and activate movement of signaling proteins that initiate MEF2-mediated transcription, however, the AKAP does not directly facilitate nuclear entry of PKD1 as the scaffold does not change localization following agonist stimulation. To investigate a possible role for Hsp20 in the nuclear influx of PKD1, we used a novel *in situ* proximity ligation assay (PLA) to allow high resolution of the distribution of the Hsp20-PKD1 complex (Figure 3). Under basal, unstimulated conditions, diffuse cytoplasmic staining of Hsp20-PKD1 complexes were observed suggesting that the Hsp20-PKD1 complex was excluded from the nucleus (Figure 3A). In contrast, Hsp20-PKD1 complexes were significantly enriched, with increased localization to the nuclei of ISO-treated cardiac myocytes (Figure 3A and B). A similar distribution pattern was also observed in cells treated with control peptide + ISO. However, treatment of the cells with disruptor peptide triggered a significant change in the intracellular localisation of Hsp20-PKD1 complexes. When the interaction between the two proteins was perturbed, significantly less Hsp20-PKD1 complex was visible globally and complexes that were observed showed little nuclear localization (Figure 3A and B). To support findings from the PLA assays and evaluate the degree of Hsp20-PKD1 complex distribution, we resorted to conventional cell fractionation (Figure 3C). In agreement with data from PLA experiments, ISO treatment triggered translocation of both PKD1 and Hsp20 to the nucleus that could be attenuated with the disruptor peptide but not with control (Figure 3D and E). PKD1 nuclear translocation during hypertrophic stimulus has been characterised previously by others [22,24,25] however, this is the first indication that the said nuclear import of PKD1 is directly mediated by another protein (in this case Hsp20).

## The Hsp20-PKD1 complex directs cardiac hypertrophy and cardiac fibrosis *in vivo*

Studies utilizing mice that have a cardiac-specific deletion of PKD1 [26] showed that PKD1 acts as a key element of the stress-stimulated signaling pathway involved in pathological cardiac remodeling. One crucial component of cardiac remodeling, contributing to compromised diastolic function, is cardiac fibrosis. Interestingly, these mice showed not only diminished cardiac myocyte hypertrophy but also significantly reduced cardiac fibrosis. This added to previous indirect evidence suggesting that PKD1 has a role in controlling cardiac fibrosis [27,28].

**Figure 4:**
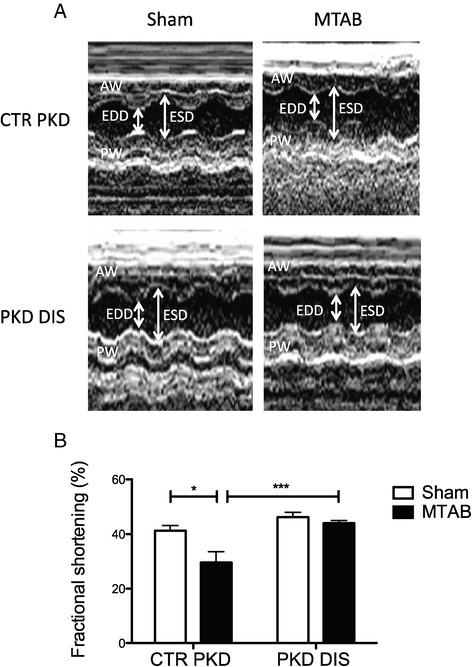
**Preserved cardiac function following Hsp20-PKD1 disruption.** Mice undergoing sham or MTAB surgery were treated with 10 mg/kg PKD-disruptor (PKD DIS) or a scrambled control (CTR PKD) peptide twice weekly for 4 weeks. **A**. Representative LV echocardiography M-mode traces; AW, anterior wall; PW, posterior; EDD, end diastolic diameter; ESD, end systolic diameter. **B**. Histogram of average ± S.E.M fractional shortening data, n ≥ 5 mice, *p < 0.05, ***p < 0.001, one-way ANOVA tests followed by post-hoc Tukey’s test.

**Figure 5:**
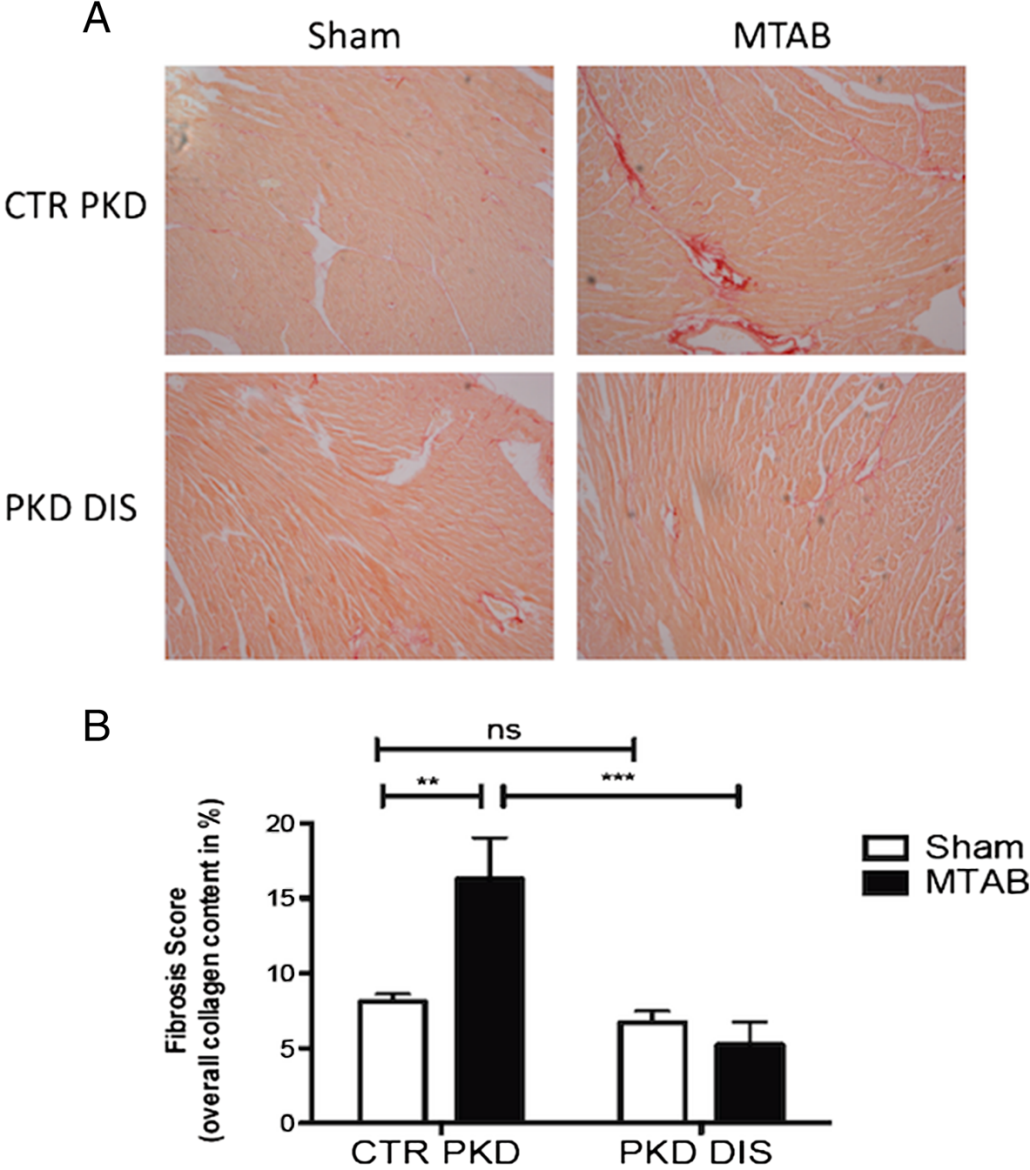
**Cardiac fibrosis is attenuated by disruption of Hsp20-PKD1 interaction. A**. Picrosirius red staining of sham and MTAB hearts following treatment of mice with 10 mg/kg PKD-disruptor (PKD DIS) or scrambled control (CTR PKD) peptide twice weekly for 4 weeks. **B**. Histogram values represent stained area expressed as a percentage of the total area of interest. Data represents mean ± S.E.M., n ≥ 3 mice, **p <0.01, ***p < 0.001, ns p > 0.05, one-way ANOVA tests followed by post hoc Tukey’s tests.

To this end, we used a minimally invasive transverse aortic banding (MTAB) model of pressure overload induced cardiac hypertrophy [29] and compared them to sham-operated mice following treatment with the Hsp20-PKD1 disruptor and control peptides. Treatment with disruptor peptide, but not control, prevented MTAB-induced decreases in left ventricular contractility (Figure 4; % fractional shortening: 44.0 ± 1.0% cf. 29.5 ± 4.0%, p < 0.0001, MTAB+DIS PKD and MTAB+CTR PKD, respectively). Analysis of picrosirius red stained heart sections from mice treated with control peptide showed significantly increased collagen deposition (fibrosis) in MTAB mice compared with sham-operated mice (Figure 5; 16.3 ± 2.8% (MTAB+CTR PKD) cf. 8.1 ± 0.5% (sham+CTR PKD), p < 0.01). In contrast, hearts from MTAB mice treated with the Hsp20-PKD1 disruptor showed significantly reduced fibrosis when compared with MTAB mice treated with control peptide (Figure 5; 5.2 ± 1.5% (MTAB+PKD DIS) cf. 16.3 ± 2.8% (MTAB control) p < 0.001). This data suggests that interdiction of the Hsp20-PKD1 complex is protective against accumulation of fibrillar collagen and development of the cardiac fibrosis that accompanies hypertrophic remodelling following pressure overload.

## Conclusions

In summary, we have discovered a novel protein complex (Hsp20-PKD1) that appears to orchestrate hypertrophy signalling pathways involved in the induction of fetal gene program, pathological cardiac growth and cardiac remodeling. As disruption of the complex significantly retards these processes, we propose, the Hsp20-PKD1 signalling axis as a promising therapeutic target to hinder the hypertrophic phenotype. Development of Hsp20-PKD1 disruptors to prevent PKD1 nuclear translocation represents a novel route for the development of anti-hypertrophic and anti-fibrotic agents.

## Methods

### Peptides

Hsp20-PKD1disruptor (GRDVAIKIIDKLRFPTKQESQLRNE) and control peptide (GAAVAIKIIAKLRFPTKQESQLRNE) were synthesised by GenScript and included an N-terminal stearoyl group (CH_3_(CH_2_)_16_COOH) making them cell permeable. For cell studies peptides were dissolved in DMSO to a stock concentration of 10 mM and used at a final concentration of 10 μM, and for *in vivo* studies were dissolved in sterile saline to 1 mg/ml.

### Antibodies

Anti-His (H1029), anti-V5 antibodies (V8137) and anti-Actinin (A7811) were from Sigma. Anti-HSP20 antibody was from Millipore (07–490). Anti-GFP (ab290), anti-phospho-HSP20 (ab58522), anti-HSP90 (ab13495) and anti-GM130 (ab52649) antibodies were from Abcam. Anti-PKD1 (sc-639) and anti-Lamin A/C (sc-56140) antibodies were from Santa Cruz. Anti-PKD1 (H00005587-A01) for immunostaining was from Abnova.

### Cell culture, transfection, Western blotting and immunoprecipitation

Neonatal rat cardiac myocytes were cultured as described before [30]. For hypertrophy induction, cells were cultured in serum-free medium for 48 h prior to stimulation by 10 μM of ISO for 24 h and where appropriate, cardiac myocytes were pretreated with compounds. DNA plasmid constructs used for transfection included V5-HSP20 in pDEST vector and GFP-PKD1 in pEF-BOS vector. HEK 293 cells were transiently transfected with both plasmid DNA using Polyfect Transfection Reagent (Qiagen) following the manufacturer’s instruction. Cellular lysates were prepared in lysis buffer [25 mM Hepes, 2.5 mM EDTA, 50 mM NaC1, 50 mM NaF, 30 mM sodium pyrophosphate, 10% (v/v) glycerol, 1% (v/v) Triton X-100, pH 7.5, containing Complete™ EDTA-free protease inhibitor cocktail tablets (Roche)] after specified treatments. Protein concentration of lysates was determined using the Bradford assay and all samples were equalised for protein concentration. Proteins were separated by SDS/PAGE (4–12% Bis-Tris gels) and transferred onto nitrocellulose membranes for Western blotting. For immunoprecipitation, HSP20 or PKD1 and V5 or GFP antibodies were used to immunoprecipitate endogenous, and over-expressed PKD1 and HSP20, respectively. The resulting immunocomplexes were captured using Protein A beads (Invitrogen) at 4°C overnight with shaking. The immunocomplexes were then collected by centrifugation at 10 000 × g for 3 min and washed three times with 3T3 lysis buffer. Bound proteins were then eluted in SDS-PAGE sample buffer and subjected to SDS-PAGE and immunoblotting. Negative controls using isotype-matched IgG (Jackson ImmunoResearch Laboratories, Inc.) from the same species as the antibodies were included to screen for non-specific binding. Immunoreactive proteins were detected using horseradish peroxidase-conjugated goat anti-rabbit or anti-mouse secondary antibody (1:5000, Sigma-Aldrich) and visualized by enhanced chemiluminescence detection (Pierce). Quantification of the band intensity was accomplished by densitometry using Quantity One 1-D software (Bio-Rad).

### ProtoArray

To prepare the probe for ProtoArray analysis, Ultimate™ ORF clone IOH57317 (Invitrogen), containing the open reading frame (ORF) of HSP20 in pENTR221 vector, was used to generate N-terminal His-fusion protein by Gateway cloning technology into pDEST-17 vector (Invitrogen). The His-tagged HSP20 protein was expressed in *E.coli* and purified by Ni-NTA Superflow resin (Qiagen). Coomassie Blue staining and Western blot with mouse monoclonal anti-His antibody (Sigma) and rabbit polyclonal anti-HSP20 antibody (Upstate-Millipore) verified the purity and specificity of the probes. Briefly, the array was incubated in blocking buffer (1×PBS, 1% BSA and 0.1% Tween 20) for 1 h at 4°C with gentle shaking to block non-specific binding. The array was then probed with 10 μM (200 μg/ml) of His-HSP20 protein in probing buffer (1×PBS, 5 mM MgCl_2_, 0.5 mM dithiothreitol (DTT), 0.05% Triton X-100, 5% glycerol, 1% BSA) for 1.5 h at 4°C. After washing, the array was incubated at 4°C with mouse monoclonal anti-His antibody (Sigma) and Alexa Fluor 647 goat anti-mouse IgG (H + L) (Molecular Probes, Invitrogen) diluted 1:2000 in probing buffer for 45 min and 30 min, respectively. After washing and drying, the array was scanned by ScanArray Express Microarray Scanner (Packard Bioscience Biochip Technology, PerkinElmer) at a wavelength of 633 nm. The results were analysed using BlueFuse for Microarrays software (BlueGnome, Cambridge), following acquisition of the ProtoArray Lot Specific information. The Confidence Flags were used to indicate the degree of confidence showing potential interactions. A control array was also included in parallel to determine probe-specific interactions and exclude any non-specific interactions.

### In vitro pull-down assay

Equal molar concentrations of purified recombinant His (negative control) or His-HSP20 and PKD1 (Abcam) were mixed in 3T3 lysis buffer and incubated end-on-end with gentle agitation for 1 hour at 4°C. Pre-equilibrated Ni-NTA Superflow resin (Qiagen) was then added to the protein cocktail and incubated with gentle agitation for another hour at 4°C. Beads were then sedimented by centrifugation at 10,000 × g for 3 min, followed by washing thrice with 3T3 lysis buffer. Proteins were then resolved by SDS-PAGE with PKD1 protein run alongside as a positive control, following by immunoblotting using an anti-PKD1 antibody.

### SPOT synthesis of peptides and overlay experiments

This was done as described by us in detail elsewhere [31].

### Immunostaining and phalloidin staining of actin

For immunofluorescent labeling, cardiac myocytes were plated onto laminin-coated 8-well chamber slides (Lab-Tek, Sigma). Cells were fixed in 95% ice-cold methanol/ 5% acetone mixture at −20°C for 10 min and permeabilised with 0.1% Triton X-100 in PBS for 10 min at room temperature. After several washes with Tris-buffered saline (TBS) (150 mM NaCl, 20 mM Tris–HCl, pH 7.6), the cells were blocked with 10% donkey serum and 2% BSA (w/v) in TBS for 2 h followed by three washes with TBS. The primary antibodies used was diluted to the required concentration in blocking buffer diluted 1:1 with TBS and added to the cells for 2 h followed by three washes with TBS. After washing, cells were incubated with 1:400 diluted secondary antibodies Alexa Fluor 488 donkey anti-mouse IgG and Alexa Fluor 594 donkey anti-rabbit IgG (Molecular Probes) for 1 h. For phalloidin staining, actin fibers were stained with Alexa Fluor 488 phalloidin (Molecular Probes, Invitrogen) diluted 1:500 in PBS for 30 min at room temperature and washed thrice in PBS. DAPI (Invitrogen) was used for nuclear counterstain. Staining was visualised using a Zeiss Pascal laser-scanning confocal microscope (LSM) 510 Meta and an Axiovert 100 microscope (Carl Zeiss, UK) equipped with an oil immersion objective (63×/1.4 NA plan apochromat lens). Single-plane images were captured at the same depth and processed on Zeiss LSM Image Examiner. For quantitative colocalisation analysis, Pearson’s (Rr), Manders’ overlap (R) and colocalisation coefficients (M1, M2) were calculated for at least 10 individual cells selected randomly using the ImageJ JACoP plugin (http://rsb.info.nih.gov/ij/plugins/track/jacop.html). M1 signifies the correlation of Hsp20 overlapping PKD1, while M2 indicates the overlap coefficient of PKD1 to HSP20. A background correction was performed prior to analysis to eliminate system variability in the image background.

### Real-time xCELLigence measurements

Cellular size of cardiac mycytes using xCELLigence technology was carried out as described previously [15]. An initial population of 40,000 cardiac myocytes/well was plated out on the laminin (BD Biosciences) pre-coated E-Plate 96 (Roche) in triplicates after background measurements were taken. Briefly after 48 h of culture in the serum-free medium, cardiac myocytes were treated with either 10 μM ISO alone or ISO following a 30 min or 2 h pre-treatment with peptides. Controls with vehicle (DMSO alone) were also performed. The cultures were continuously monitored for up to 48 h and the impedance as reflected by cell index (CI) values was set to record every 30 min. The xCELLigence data were then analysed using the RTCA software (Roche Applied Science). The results were expressed by normalized CI, which are derived from the ratio of CIs before and after the addition of compounds.

### Measurement of protein content

Cardiac myocytes were plated on six-well plates and cultured in a serum-free condition for 48 h before experiments. After stimulation of cardiac myocytes (2 h of compound + 24 h of ISO or 24 h of ISO alone), each well was rinsed three times with cold PBS. The cells were then scraped with 1 ml of 1× standard sodium citrate (SSC) containing 0.25% (w/v) SDS and vortexed extensively. The total cell protein and the DNA content were determined according to the manufacturers’ instructions using Total Protein Kit (Sigma #TP0200) and DNA Quantitation Kit (Sigma #DNA-QF), respectively. The protein content was normalised by the DNA amount to correct for differences in the cell number.

### Quantitative real-time PCR analysis of fetal gene expression

Total RNA of each condition was extracted using a TRIzol reagent (Invitrogen) and purified with the RNeasy Mini Kit (Qiagen). One microgram of DNase-digested total RNA was reverse-transcribed to first strand complementary DNA (cDNA) using AffinityScript Multiple Temperature cDNA synthesis kit (Agilent, Edinburgh, UK) according to manufacturer’s instructions. Real-time PCR primer-probe sets (Eurofins MWG operon) were design assisted by the Primer 3 software. Primer-probe sets were as follows: Atrial natriuretic peptide (ANP): 5′-AGGCTGCAACAGCTTCCGGT-3′ (probe), 5′-GGATTGGAGCCCAGAGCGGAC-3′ (sense), 5′-CGCAAGGGCTTGGGATCTTTTGC-3″ (antisense); Brain natriuretic peptide (BNF): 5′-GCTGCTGGAGCTGATAAGAGAAAAGT-3′ (probe), 5′-AGCCAGTCTCCAGAACAATCCACG-3′ (sense), 5′-AGGGCCTTGGTCCTTTGAGAGC-3′ (antisense); β-myosin heavy chain (β-MHC): 5′- CTGGATGAGGCAGAGGAGAG-3′ (probe), 5′-CCAACACCAACCTGTCCAA-3′ (sense), 5′-CAGCTTGTTGACCTGGGACT-3′ (antisense) and rat 18S rRNA: 5′- TGAGGCCATGATTAAGAGGG-3′ (probe), 5′-CGCGGTTCTATTTTGTTGGT-3′ (sense), 5′-CGGTCCAAGAATTTCACCTC-3′ (antisense). Rat 18S rRNA was used as an internal control for normalising relative expression levels in the different samples. Real-time PCR reactions were prepared using Platinum® Quantitative PCR SuperMix UDG with ROX (Invitrogen) and performed in a 7300 Real time PCR System (Applied Biosystems). Each PCR amplification was run in triplicate using the following conditions: 2 min at 95°C, followed by a total of 40 cycles (15 s at 95°C and 1 min at 60°C). Relative gene expression was calculated using the comparative threshold method (2^-∆∆Ct^) and is presented as fold change of transcripts for gene of interest compared to 18 s rRNA.

### Duolink™ proximity ligation assay

PKD1 association with HSP20 was investigated further by using the Duolink*™* Proximity Ligation Assay (PLA) (Olink Bioscience, Uppsala, Sweden). After fixation, permeabilisation and blocking, slides were incubated with primary antibodies against PKD1 and HSP20 raised in two different species. Then PLA probes, which are conjugated with oligonucleotides, were introduced to recognise the primary antibodies. A solution that promotes hybridization between the PLA oligos was then added, where a hybridisation reaction only occurred if the two proteins were in close proximity (<40 nm), but not if they were far apart. This reaction was followed by ligation of the oligonucleotides and a rolling circle amplification (RCA) reaction, where a repeated sequence product was made. This product was then detected using fluorescently labeled oligonucleotides, where a Hsp20-PKD1association appeared as discrete red dots under the microscope. Cells were counterstained with α-actinin to show sarcomeric stained. Slides were finally mounted under coverslips with DUOLINK mounting media and visualised. In order to detect all PLA signals, a series of Z-stack images was collected.

### Subcellular fractionation of cardiac myocytes

Cardiac myocytes were fractionated into cytosolic, membrane and nuclear fractions using FractionPrepTM (BioVision, Mountain View, CA) following the manufacturer’s protocol. Equal amounts of each cell lysate were used for Western blot analysis. To assess the purity of fractionation, cytoplasmic, membrane and nuclear fractions were confirmed by immunoblotting using anti-HSP90 as cytoplasmic marker, anti-GM130 as membrane marker and anti-Lamin A/C as nuclear marker, respectively.

### Minimally invasive transverse aortic banding and transthoracic echocardiography

Healthy adult male C57BL/6 J mice (Harlan, UK) weighing between 25-30 g were used for these experiments. Procedures conformed to the UK Animals (Scientific Procedures) Act 1986 and were approved by institutional ethical review committees. MTAB and sham surgical protocols were performed as previously described [29]. Immediately following surgery, animals were injected (intraperitoneal) with either Hsp20-PKD1disruptor peptide or a scrambled control peptide at 10 mg/kg twice weekly and were left for 4 weeks to allow cardiac remodeling to occur. Echocardiographic assessment of left ventricular (LV) function was performed 4 weeks after MTAB or sham surgery, as described previously [29]. LV end systolic dimension (LVESD) and LV end diastolic dimension (LVEDD) were assessed from M-mode traces and % fractional shortening calculated. Fractional shortening (FS) is expressed as [(LVEDD-LVESD)/LVEDD] ×100. An average of three measurements of each variable per animal was used.

### Picrosirius red staining

Four weeks post-surgery, animals were euthanized with an intravenous injection of pentobarbital sodium (Euthatal, 200 mg/kg). Terminal anaesthesia was confirmed by testing loss of the pedal reflex. Hearts were rapidly excised and washed thoroughly in ice-cold Ca^2+^-free Krebs solution (120 mM NaCl, 5.4 mM KCl, 0.52 mM NaH_2_PO4, 20 mM HEPES, 11.1 mM glucose, 3.5 mM MgCl_2_, 20 mM Taurine, 10 mM Creatine; pH 7.4). LV tissue was processed, embedded in paraffin and sectioned. Slides were stained for 1 h in picrosirius red solution containing 0.1% (w/v) direct red 80 dye in a saturated aqueous solution of 1.3% picric acid. Five random sections per heart (5 μm sections with a distance of 200 μm between sections), and 5 areas of interest per section were photographed at 10× magnification using non-polarised light with a Leica DM LB2 microscope and a Leica DFC 320 camera (Leica Microsystems, Germany). Quantification of picrosirius red staining used ImageProPlus software (version 5.0; MediaCybernetics), with stained area expressed as a percentage of the total area of interest. Values were averaged to give one representative value per heart. For calculation of threshold, the intensity of signal for background was set using unstained area of tissue.

### Statistical analysis

Values are presented as mean ± S.E.M. from at least three independent experiments. Statistical significances between groups were determined by the use of Student’s *t*-test or one-way ANOVA tests followed by post hoc Tukey’s tests. Values were considered significant if *p* < 0.05. Where representative immunoblots or immunocytochemistry images were shown, similar data were obtained n ≥ 3 times.
